# Ultrafast Widefield Mid-Infrared Photothermal Heterodyne Imaging

**DOI:** 10.1021/acs.analchem.2c02548

**Published:** 2022-10-05

**Authors:** Eduardo M. Paiva, Florian M. Schmidt

**Affiliations:** Department of Applied Physics and Electronics, Umeå University, SE-90187Umeå, Sweden

## Abstract

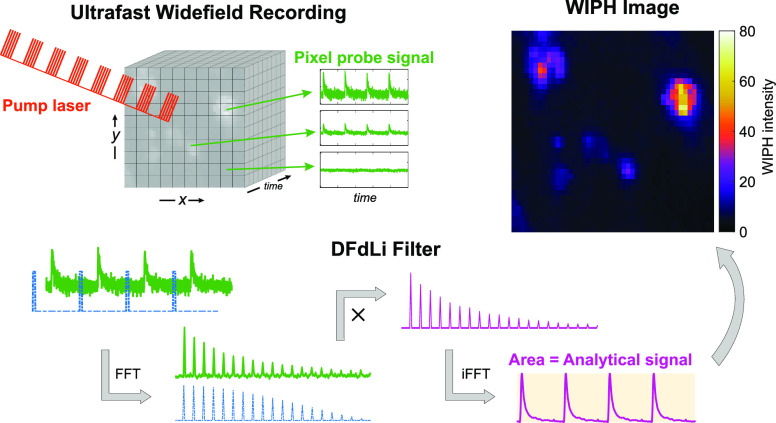

Mid-infrared photothermal (MIP) microscopy is a valuable tool for sensitive and fast chemical imaging with high spatial resolution beyond the mid-infrared diffraction limit. The highest sensitivity is usually achieved with heterodyne MIP employing photodetector point-scans and lock-in detection, while the fastest systems use camera-based widefield MIP with pulsed probe light. One challenge is to simultaneously achieve high sensitivity, spatial resolution, and speed in a large field of view. Here, we present widefield mid-infrared photothermal heterodyne (WIPH) imaging, where a digital frequency-domain lock-in (DFdLi) filter is used for simultaneous multiharmonic demodulation of MIP signals recorded by individual camera pixels at frame rates up to 200 kHz. The DFdLi filter enables the use of continuous-wave probe light, which, in turn, eliminates the need for synchronization schemes and allows measuring MIP decay curves. The WIPH approach is characterized by imaging potassium ferricyanide microparticles and applied to detect lipid droplets (alkyne-palmitic acid) in 3T3-L1 fibroblast cells, both in the cell-silent spectral region around 2100 cm^–1^ using an external-cavity quantum cascade laser. The system achieved up to 4000 WIPH images per second at a signal-to-noise ratio of 5.52 and 1 μm spatial resolution in a 128 × 128 μm field of view. The technique opens up for real-time chemical imaging of fast processes in biology, medicine, and material science.

## Introduction

Optical microscopy combined with vibrational spectroscopy has become an essential tool for chemical imaging in life science.^1^ The ability to visualize biomolecules noninvasively in living cells contributes to our understanding of cell metabolism and signaling and thereby improves medical diagnostics and therapy.^2^ The most established far-field imaging techniques, Raman, fluorescence, and infrared (IR) absorption, typically excel in spatial resolution, sensitivity, or speed^3^ and are usually limited to image acquisition times of minutes or tens of seconds at most, due to long averaging times and/or point-scanning schemes.^2^ However, some important biological processes, such as the cellular uptake of free fatty acids (FFAs),^4^ cell signaling via gasotransmitters,^5,6^ and the germination of bacterial spores,^7^ may occur on a subsecond time scale.

While near-field vibrational techniques, e.g., those coupled to atomic-force microscopy, offer superior spatial resolution, most imaging applications in biological and medical research employ far-field Raman microspectroscopy. This technique can resolve internal cell structures and, unlike fluorescence, can be label-free and is not hampered by photobleaching. Advanced Raman techniques, such as stimulated Raman scattering, have pushed the spatial resolution down to ∼130 nm and enable submillisecond pixel dwell times,^1^ but the small scattering cross sections (σ_S_ ∼ 10^–30^ cm^2^ sr^–1^),^8^ the subsequent long averaging times, and the constraints to point-scan strategies slow down image acquisition to the extent that it precludes real-time monitoring of fast biological processes in a large field of view (FoV). Fourier-transform infrared microspectroscopy (μFTIR) lacks the necessary spatial resolution, due to the inherent diffraction limitation at the long wavelengths. Thus, one challenge is to simultaneously achieve high sensitivity, spatial resolution, and speed in a large field of view (FoV).^9^

**Figure 1:**
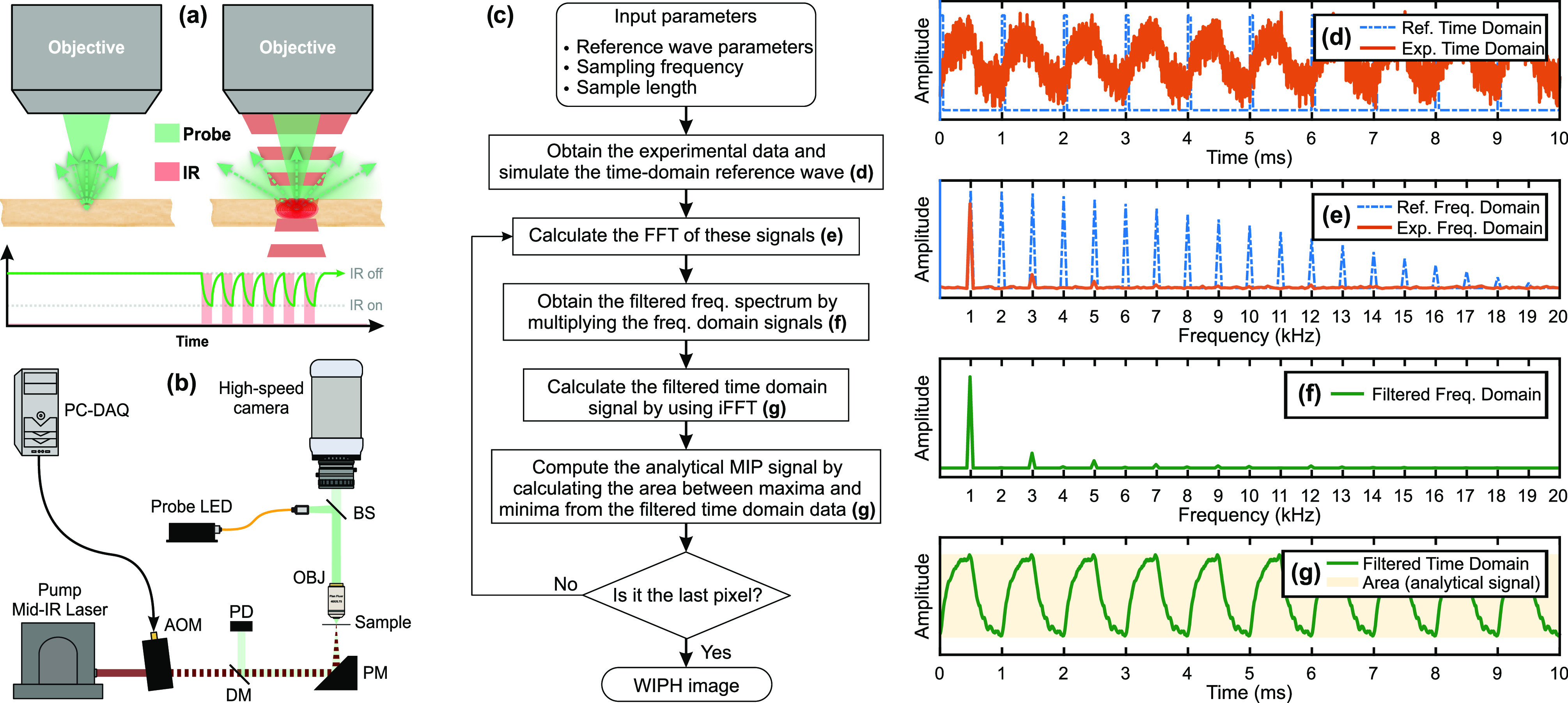
(a) Principle of WIPH using cw probe light, whose intensity decays due to scattering following thermal excitation with a mid-IR pump laser. (b) Schematic drawing of the WIPH microscope setup including the mid-IR pump laser (2015–2220 cm^–1^), acousto-optic modulator (AOM), visible probe light-emitting diode (LED, 617 nm), high-speed CMOS camera, and CaF_2_ sample slide. OBJ, 40 × 0.75 objective lens; PD, photodetector; DM, germanium dichroic mirror; PM, parabolic mirror; BS, beam splitter. (c) Flow-chart of the digital frequency-domain lock-in (DFdLi) filter. (d–g) Graphs illustrating the main steps of the DFdLi filter, as explained in (c). (d) Simulated MIP time domain signal (solid line) and simulated square wave reference signal at 5% duty cycle (dashed line). (e) Frequency spectra of the signals in (d) after applying an FFT. (f) Result of multiplying the frequency spectra in (e). (g) Filtered time-domain signal after applying an inverse FFT on the data in (f). The shaded area is used to compute the analytical WIPH signal.

Recently, a novel pump-probe technique, mid-IR photothermal (MIP) imaging, has been introduced,^10−14^ where modulated light from a mid-IR pump laser in resonance with vibrational transitions of the molecular target species induces periodic temperature changes in the sample. The temperature variations, in turn, cause local changes in the sample refractive index, which can be detected by visible, fixed-frequency probe light (Figure 1a), thereby pushing the diffraction limit to ≤1 μm, similar to Raman microscopy, while maintaining the high sensitivity enabled by the large mid-IR absorption-cross sections.

The most sensitive MIP variant, IR photothermal heterodyne imaging (IR-PHI) usually employs a high-bandwidth photodetector and a phase-sensitive lock-in amplifier (LIA) to extract the pump laser modulation frequency in the probe light and obtain point-scan images. Li et al.^10^ demonstrated super-resolution point-scan IR-PHI imaging with a 300 nm spatial resolution using a laser emitting at 530 nm and a high numerical aperture (NA) objective. Using 30 ms pixel dwell time, it took approximately 1 min to record one IR-PHI image with 5 × 5 μm FoV.

The MIP modulation commonly has a low duty cycle, which increases the signal strength of the higher harmonics at the expense of the lower harmonics. Since a LIA usually demodulates only one of the (lower) harmonics, the MIP signal amplitude is not maximized, and the nonlinear photothermal decay cannot be accurately resolved. This led to the development of multiharmonic demodulation MIP schemes, such as photothermal dynamics imaging (PDI).^15^ PDI makes use of high bandwidth detection to obtain nanosecond temporal resolution per pixel within a single pulse excitation and enables retrieval of the thermal decay profile, which can improve the chemical contrast in complex samples and provide valuable physical properties of the target. The PDI technique demonstrated improved imaging speed and signal-to-noise ratio (SNR) compared to LIA detection but still had the drawback of point-scanning tied to the limited scan speed of the sample holder *x*–*y* stage.

To overcome the speed limitation of point-by-point scanning, camera-based widefield MIP has been developed employing brightfield^16^ and darkfield^17^ intensity detection, as well as phase-sensitive methods.^18−20^ A brightfield microscope was developed by Bai et al.^16^ based on a virtual lock-in scheme requiring precise synchronization of the pump and probe pulses with each other and with the camera exposure trigger. The system enabled MIP imaging of a poly(methyl methacrylate) (PMMA) film at 1250 frames/s with a spatial resolution of 510 nm and an SNR of 2. The authors applied the microscope to imaging of lipid droplets in living SKOV3 human ovarian cancer cells at an image acquisition rate of 2 Hz. Similar virtual lock-in camera approaches were subsequently used in combination with other imaging techniques.^17,21,22^

Zong et al.^17^ developed a darkfield MIP microscope with a 70 × 70 μm FoV using pupil engineering to suppress the brightfield background light. The pupil blocker provided 3 orders of magnitude background suppression and a 6-fold SNR improvement compared to brightfield detection, as well as an improved sensitivity to subwavelength particles. The microscope was used to image bacteria and lipid droplets in cancerous cells at 400 frames/s. However, the low intensity of the darkfield radiation required extensive averaging, thus sacrificing speed. In a different approach, Toda et al.^18^ implemented widefield MIP detection by molecular contrast on phase contrast (MC-PC) microscopy. Here, the imaged phase-contrast was modulated by the MIP excitation. The authors demonstrated detection of silica and polystyrene microbeads at 3000 frames/s with a spatial resolution of 1 μm and an SNR of 13.2. The MC-PC signal was obtained by calculating the difference between two consecutive images that represent the mid-IR pump laser on and off states.

In this work, we combine camera-based MIP with heterodyne MIP to enable widefield mid-IR photothermal heterodyne (WIPH) imaging with high speed, sensitivity, and spatial resolution using a brightfield microscope. An ultrafast CMOS camera recording at >100 k frames/s is employed such that each pixel essentially acts as a photodetector. WIPH is realized without the need for synchronization by using continuous wave (cw) probe light and a novel digital frequency-domain lock-in (DFdLi) filter. The DFdLi filter simultaneously extracts multiple harmonics of the pump laser modulation frequency from the probe signals recorded by the camera pixels, thereby effectively suppressing the noise at all other frequencies.

The performance of the WIPH technique is assessed by imaging potassium ferricyanide (K-FeCy) powder in the cell-silent spectral region around 2100 cm^–1^. MIP spectra of K-FeCy acquired using the DFdLi filter are compared to IR-PHI spectra measured with a commercial LIA, FTIR spectra, and WIPH spectral data. The SNR is assessed as a function of probe power, integration time, and number of demodulated harmonics, and typical photothermal decays constants for ferricyanide particles are determined. Finally, the WIPH system is applied to image alkyne-tagged fatty acids in a living 3T3-L1 fibroblast cells.

## Experimental Section

### Widefield Mid-IR Photothermal Heterodyne Imaging System and Data Acquisition

A schematic drawing of the experimental WIPH setup is shown in Figure 1b. A cw external-cavity quantum cascade laser (EC-QCL, TLS-41047-MHF, Daylight solutions) operating in the spectral range from 2015 to 2220 cm^–1^ was used as a mid-IR pump source. The EC-QCL had a peak output power of 160 mW and a full-width at half-maximum (FWHM) of <10 MHz. An acousto-optic modulator (AOM, M1208-G80-MIR, Isomet) was employed to impose a square wave modulation (200–4000 Hz) on the pump light. In the case of live cell measurements, a combination of a fast modulation at 100 kHz and a slow modulation at 500 Hz was employed to reduce the average power impinging onto the sample.

The probe light at 617 nm was provided by a light-emitting diode (LED, LEDMOD.V2, Omicron Laserage) with 15 nm FWHM and maximum output power of 450 mW. An optical fiber (M107L01, Thorlabs) with fiber-collimator (F950SMA-A, Thorlabs) was used to couple the LED beam to the microscope. Images were recorded with an ultrafast CMOS camera (FASTSCAN NOVA S16, Photron) that featured a speed of 16 k frames/s at full frame (1024 × 1024), and up to 1.1 million frames/s at reduced frame size. The camera had a quantum efficiency of 78% at 590 nm, a minimum exposure time of 0.2 μs, a pixel size of 20 μm, and 128 Gb of internal fast memory, which is important as tens of thousands of frames are usually recorded for one WIPH image.

An objective (N40X-PF, Thorlabs) with a magnification of 40× and NA of 0.75 and a 200 mm focal length tube lens (AC254-200-A, Thorlabs) were used to focus the image onto the camera sensor. The probe optical power at the camera sensor, needed to calculate the power per pixel for the SNR evaluation, was measured with the help of a photodiode power meter (S130C, Thorlabs). The probe power needed to saturate a camera pixel was 1.64 nW. The waveform sent to the AOM for pump laser modulation was generated by a data acquisition board (PCIe-6376, National Instruments) using LabVIEW.

A counter-propagating microscopy scheme was used (Figure 1b), in which the visible probe beam was focused onto the sample from above by the objective, whereas the IR pump beam was intentionally poorly focused from below using a 90° off-axis parabolic mirror (MPD129-P01, Thorlabs). A beam-splitter (CM1-BP145B1, Thorlabs) was used to pick off the reflected probe light to be detected by the camera. At the same time, the transmitted probe light was directed to a photodiode (PDA36A-EC, Thorlabs) using a dichroic mirror made of germanium (WG91050-C9, Thorlabs). The system thus allows easy conversion to transmission imaging by switching the probe fiber tip and photodetector positions. The optical spatial resolution of the acquired images was determined using a standard NBS 1952 test target, and the WIPH spatial resolution was measured with the help of isolated sample particles.

MIP spectra of potassium ferricyanide (K-FeCy) and alkyne-palmitic acid (alkyne-PA) particles were acquired with the photodetector by tuning the EC-QCL over its entire operational wavelength range, which took approximately 27 s. The wavenumber scale was obtained using a germanium etalon (OP-5483-50.8, LightMachinery) with a free-spectral-range of 734.2 MHz. To implement IR-PHI, a commercial phase-sensitive lock-in amplifier (LIA, SR830 DSP, Stanford Research Systems) was employed. The LIA integration time was 3 ms. A digital frequency-domain lock-in filter built in MATLAB, here denoted DFdLi, was applied to extract the MIP signals and WIPH images. In a separate measurement, a reference IR spectrum of K-FeCy was acquired using a diffuse reflectance FTIR spectrometer (IFS66v/S, Bruker) with 2 cm^–1^ spectral resolution in the range 400 to 4000 cm^–1^ and averaging 100 scans per spectrum.

### Digital Frequency-Domain Lock-in Filter

A flow-chart of the DFdLi filter operating principle is shown in Figure 1c. The filter workflow is further illustrated in Figure 1d–g, where simulated MIP signals before and after filter application are displayed. The DFdLi filter employs a fast Fourier transform (FFT) algorithm to convert the transient time-domain MIP signals recorded by the camera pixels, and a simulated reference wave signal (Figure 1d), to the frequency-domain (Figure 1e). Then, the two frequency-domain vectors are multiplied to extract solely the desired frequencies, i.e., harmonics of the IR pump modulation frequency, consequently eliminating the noise at all other frequencies (Figure 1f). The result is independent of the phase between experimental and reference signal.

The inverse FFT (iFFT) is then employed to calculate the filtered time-domain signal (Figure 1g). We further define the analytical WIPH signal per pixel as the area between the maxima and minima of the filtered time-domain signal (shaded area in Figure 1g). In this way, the WIPH signal does not depend on fluctuations of the DC background level, only on the amplitude of the modulated signal, which is proportional to the molecular absorption cross section of the target species.^23^

The reference waves were designed digitally in MATLAB and must have the same frequency as the IR pump laser modulation. Different types of reference waveforms can be used depending on the desired harmonics to be extracted. A single sine wave will extract only one of the harmonics, while a sum of sine waves will demodulate several specific harmonics. A square wave will extract all harmonics, which is important when the aim is to recover thermal MIP decay profiles or maximize the SNR. The square wave duty cycle determines the relative weight of the higher harmonics in the signal evaluation.

For WIPH image acquisition with the CMOS camera, the EC-QCL was kept at a fixed wavelength and camera frames were acquired continuously with rates between 50k and 200k Hz. The raw data cube then contained the 2D spatial dimensions (*x* and *y* directions) and the time dimension (*z* direction), corresponding to the modulated MIP signal for each pixel. Finally, the DFdLi filter was applied to the data cube to extract the WIPH image. The data processing workflow was as follows: (1) unfold the 3D data into a 2D matrix by stacking the pixels along the matrix rows, (2) apply the DFdLi filter per pixel/row to obtain a column vector containing the analytical signal per pixel/row, and (3) refold the column vector with the same spatial dimensions (*x* and *y*) as the initial data to obtain the WIPH image.

The instrumental parameters employed in the imaging experiments presented in this work are summarized in Table 1.

**Table 1:** Instrumental Parameters for the Imaging Experiments Carried out in this Work

exp.\parameter	wavelength (cm^–1^)	pump freq. (kHz)	duty cycle (%)	FOV (μm)	frame rate (frames/s)	no. of frames	integration time (ms)	reference wave
exp. A (Figure 3)	several	2	25	128 × 128	100 k	10 k	100	sine
exp. B (Figure 4)	2116	0.5	50	64 × 64	100 k	20 k	200	sine
exp. C (Figure 5)	2116	4	50	64 × 64	200 k	10 k	0.25–50	sine
exp. D (Figure 6)	2116	0.2	2	128 × 128	150 k	7.5 k	50	multiple sines
exp. E (Figure 7)	2116	0.2	2	128 × 128	150 k	7.5 k	50	square
exp. F (Figure 8)	2120	0.5 + 100	50	64 × 64	50 k	20 k	400	sine (0.5 kHz)

### Samples

Potassium ferricyanide was chosen as target chemical species to characterize and validate the WIPH system, since the compound exhibits narrow absorption features with large absorption cross sections in the operating range (cell-silent window) of the EC-QCL pump laser. Potassium ferricyanide powder (702587, Merck/Sigma-Aldrich) with an average particle size of 4 μm was suspended in 1-propanol and sandwiched between two CaF_2_ plates and a 15 μm Teflon spacer.

For the lipid droplet measurements, a solution containing 3T3-L1 fibroblast cells was released over a CaF_2_ glass slide. The cells were left to starve for 1 h using serum-free media and then incubated with an alkyne-PA solution for 30 min, washed, and fixed for 30 min using 4% paraformaldehyde.^24^ During incubation, the alkyne-PA was taken up by the cells and formed lipid droplets.

## Results

### Photothermal Spectra of Potassium Ferricyanide around 2100 cm^–1^

**Figure 2:**
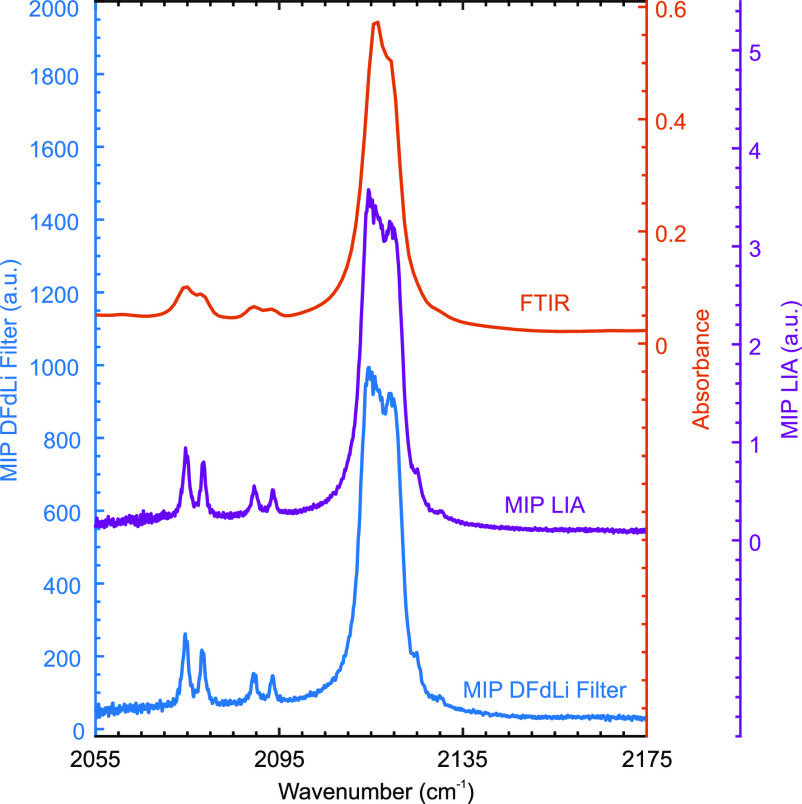
IR spectra of potassium ferricyanide recorded with FTIR, IR-PHI (MIP with LIA detection), and MIP employing the DFdLi filter.

Figure 2 shows photothermal spectra of K-FeCy acquired with the photodetector and using the LIA as well as the DFdLi filter. The IR pump modulation frequency was 2 kHz, and the MIP spectra were normalized by the power spectrum of the EC-QCL. The corresponding FTIR spectrum is shown as a reference. As expected, the MIP signal amplitude was proportional to the sample absorption cross section. The raw data contained a DC background, but the modulation is only present if the pump laser is in resonance with the target molecules, as illustrated by the fact that the MIP signal amplitude drops to almost zero off resonance. Thus, the DC background did not contribute to the analytical MIP signal.

There is an excellent agreement between the three spectra shown in Figure 2. The MIP spectra exhibit a higher spectral resolution (limited only by the laser linewidth) than the FTIR measurement, which is limited by the spectrometer resolution. As a result, the four small peaks between 2070 and 2100 cm^–1^ can be better resolved with MIP. The fact that the MIP spectrum recorded with the DFdLi filter is very similar to the IR-PHI spectrum shows that the application of the DFdLi filter is comparable to using a LIA.

### Widefield Mid-Infrared Photothermal Heterodyne Imaging

To enable WIPH imaging, it was first validated that the MIP spectrum presented in Figure 2 can be obtained from the camera pixels by applying the DFdLi filter. MIP raw data cubes were acquired at nine fixed wavenumbers of 2074.5, 2080, 2100, 2112, 2116, 2121, 2125, 2140, and 2160 cm^–1^. The instrumental parameters for this acquisition are shown in Table 1 (exp. A).

**Figure 3:**
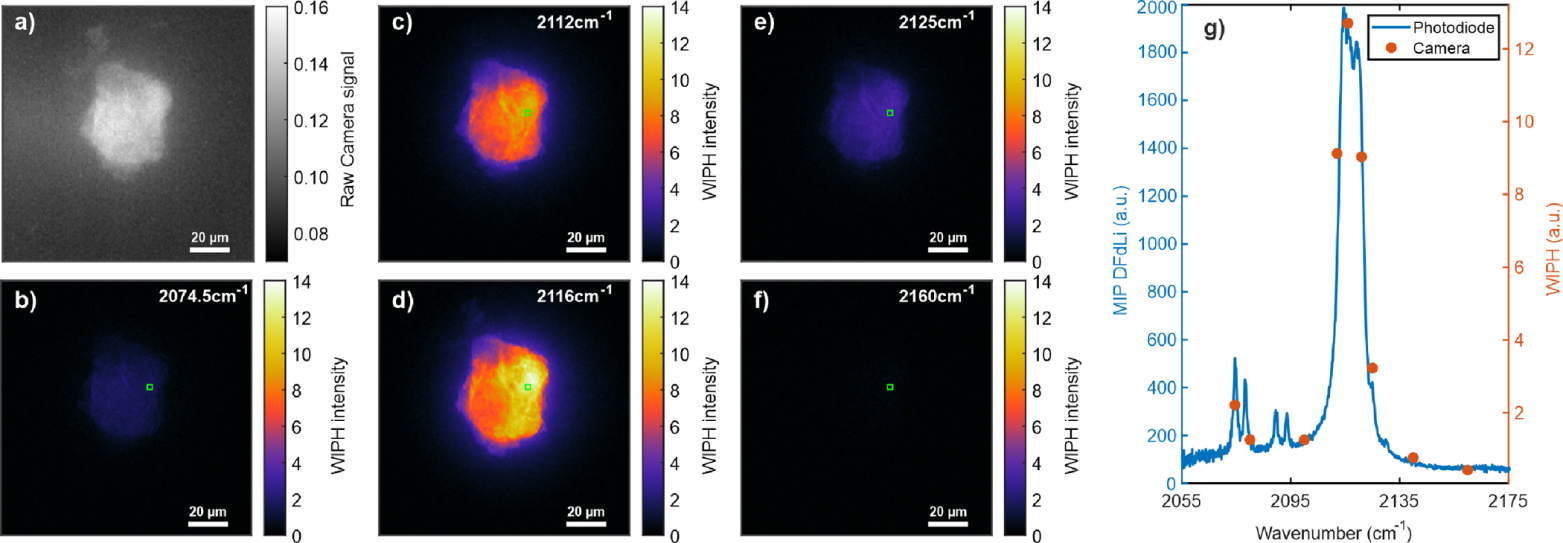
(a) Raw camera and (b–f) WIPH images of a potassium ferricyanide particle at wavenumbers 2074.5, 2112, 2116, 2125, and 2160 cm^–1^. Scale bar: 20 μm. (g) MIP spectrum of ferricyanide acquired with a photodetector and DFdLi (solid line) and nine WIPH data points (circular markers) at 2074.5, 2080, 2100, 2112, 2116, 2121, 2125, 2140, and 2160 cm^–1^. The WIPH data points represent an average of 25 pixels highlighted as green squares in the WIPH images (b–f).

The DFdLi filter was applied per pixel along the time-domain dimension to extract the WIPH image for each wavelength. The raw camera image and five of the WIPH images, for the wavenumbers 2074.5, 2112, 2116, 2125, and 2160 cm^–1^, are shown in Figure 3a–f. An average of the signal from 25 pixels, as highlighted by the green square in the WIPH images, was then used to map out the WIPH spectrum (circular, orange markers) in Figure 3g. The WIPH spectral data was consistent with the spectrum acquired using the photodetector and DFdLi filter (solid, blue line in Figure 3g), confirming the high spectral fidelity and chemical contrast of WIPH.

### Signal-to-Noise Ratio as a Function of Probe Power, and WIPH Spatial Resolution

**Figure 4:**
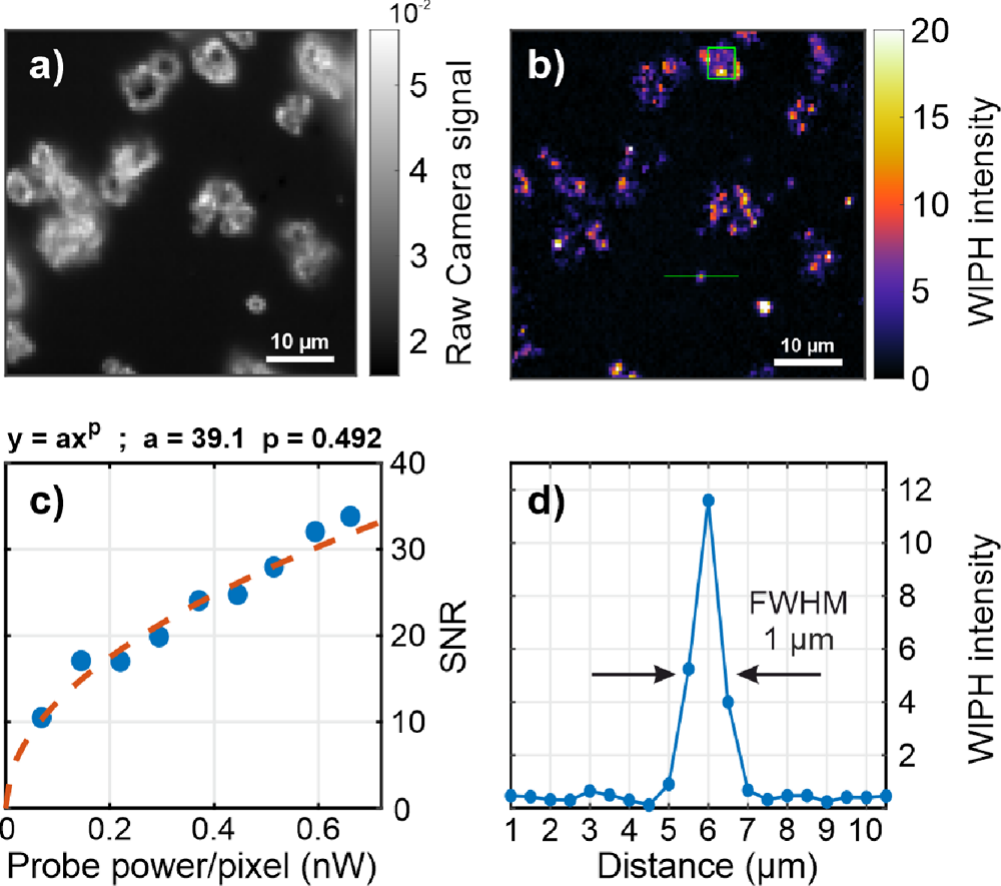
(a) Raw camera image of ferricyanide microparticles recorded with a probe power of 0.66 nW per pixel. (b) WIPH image of (a). Scale bar: 10 μm. (c) Plot of the SNR obtained from the nine WIPH images (blue dots) against probe power, together with a power function fit revealing an exponent of 0.492, which indicates shot-noise-limited detection. The SNR data points represent an average of 100 pixels highlighted as the green square in the WIPH image. (d) Plot of the pixel intensities along the green line in (b) showing the WIPH spatial resolution of 1 μm.

Figure 4 presents the results from an evaluation of the SNR as a function of the average probe power per pixel and the WIPH spatial resolution. Figure 4a displays the raw camera image of several K-FeCy microparticles in the size range from 1 to 15 μm, while Figure 4b shows the corresponding WIPH image, both recorded at the highest probe power tested. The experimental parameters for these measurements are provided in Table 1 (exp. B).

The SNR in Figure 4c, was calculated as the ratio of the average intensity of 100 pixels within the green square highlighted in Figure 4b, at IR laser modulation, and the standard deviation of the intensities for the same pixel area and integration time but without using an IR laser. This area covers one particle. The evaluated probe power values per pixel inside the green square were 0.07, 0.14, 0.22, 0.29, 0.37, 0.44, 0.51, 0.59, and 0.66 nW in average, and the obtained SNR values were 10.5, 17.1, 17.0, 19.9, 24.1, 24.8, 27.9, 32.0, and 33.8, respectively.

A power function was curve-fitted to the experimental data, revealing an exponent of 0.492, which indicates close-to shot-noise limited detection and, thus, revealing that the contribution of systematic and 1/*f* noise was low.^22^ This fitting performance is in accordance with the theoretical SNR equation for heterodyne MIP, which assumes an ideal detector and shot-noise-limited detection, and can be written as^25^
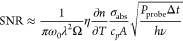
1where ω_0_ is the probe beam focal radius, λ is the probe light wavelength, Ω is the angular modulation frequency, ∂*n*/∂*T* is the thermorefractive coefficient of the medium, σ_abs_ is the absorption cross section, *C_p_* is the heat capacity per unit volume of the photothermal medium, *A* is the diffraction-limited area of the pump beam, *P*_probe_ and *h*ν are the probe power and photon energy, respectively, and Δ*t* is the integration time.

As shown in Figure 4d, which displays the line intensity of the pixels along the green line in Figure 4b, the effective spatial resolution of the WIPH image system was ≤1 μm.

### Signal-to-Noise Ratio as a Function of Integration Time and WIPH Temporal Resolution

**Figure 5:**
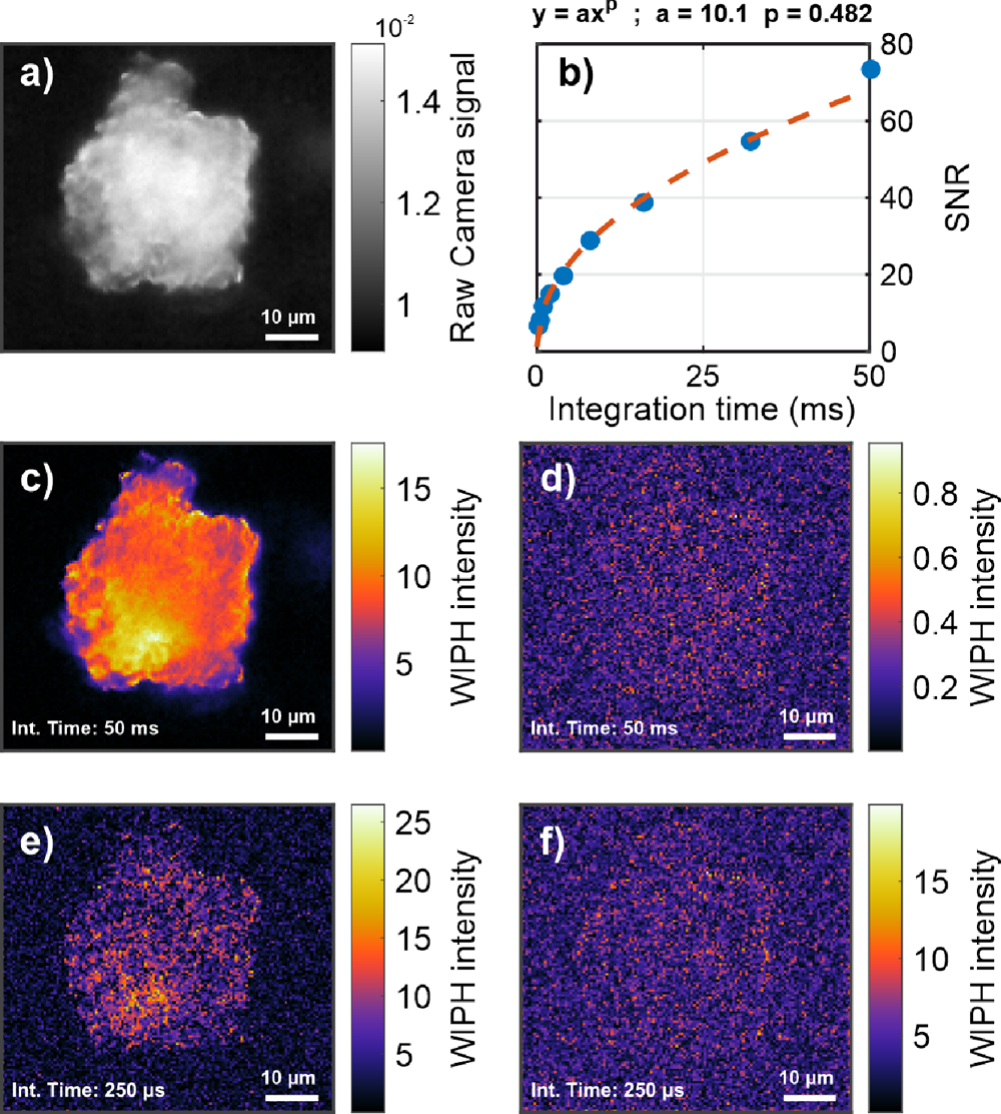
(a) Raw camera image of a ferricyanide grain containing several aggregated microparticles. (b) Plot of the SNR obtained from the nine WIPH images (blue dots) as a function of integration time, together with a power function fit (exponent of 0.482). (c) and (d) are WIPH images with and without IR laser modulation, respectively, employing 50 ms of integration time (20 Hz). (e) and (f) are WIPH images with and without IR laser modulation, respectively, employing 250 μs of integration time (4 kHz).

Figure 5 presents the results from an evaluation of the SNR as a function of integration time, as well as the temporal resolution capability. WIPH images were obtained for an agglomeration of K-FeCy microparticles using the instrumental parameters given in Table 1 (exp. C).

Nine WIPH images were obtained using the DFdLi filter and restricting the integration times to 0.25, 0.5, 1, 2, 4, 8, 16, 32, and 50 ms (Figure 5), which corresponds to 4000, 2000, 1000, 500, 250, 125, 62.5, 31.125, and 20 chemical WIPH images per second, respectively. The SNR was calculated as the ratio of the average intensity of 100 pixels in the central part of the particle with IR pump on (e.g., Figures 5c,e, with integration times of 50 and 0.25 ms, respectively) and the standard deviation of the intensities for the same pixel area and integration time but not using the IR pump (e.g., Figures 5d,f, with integration times of 50 and 0.25 ms, respectively). The SNR values obtained for the above-mentioned integration times were 5.52, 6.95, 10.6, 13.8, 18.5, 27.7, 37.5, 53.4, and 72.1, respectively. These results represent a power function with an exponent of 0.482 (Figure 5b), again in accordance with eq 1.

### Signal-to-Noise Ratio as Function of the Multiharmonic Demodulation

**Figure 6:**
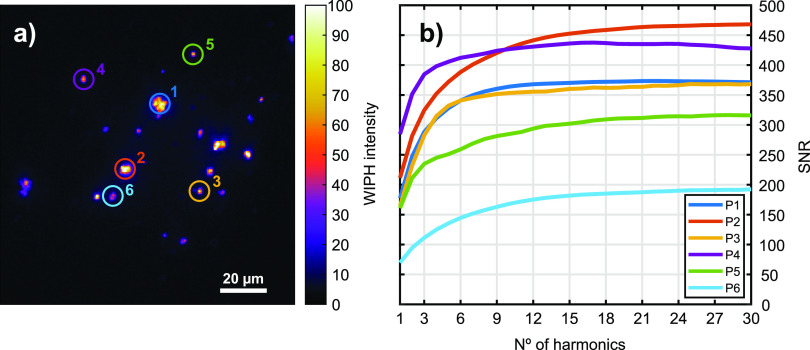
(a) WIPH image of several ferricyanide microparticles in a 128 × 128 μm field of view. (b) Plot of the SNR obtained from the six microparticles highlighted in (a) as a function of the number of harmonics demodulated with the DFdLi filter.

The SNR of the WIPH-DFdLi configuration was also evaluated as a function of the number of demodulated harmonics and using K-FeCy particles of different sizes as a sample. Figure 6a shows a WIPH image of several K-FeCy microparticles, and Figure 6b displays the SNR for six of the microparticles, highlighted in Figure 6a, plotted against the number of evaluated harmonics. Here, the sum of multiple sine waves was used as reference signal to select the desired number of harmonics. The instrumental parameters are again shown in Table 1 (exp. D). Calculating the ratio between the SNR values obtained with the first frequency (fundamental) and the first 30 frequencies (fundamental +29 harmonics), SNR improvements of 2.09, 2.21, 2.24, 1.50, 1.97, and 2.76 were obtained for particles P1, P2, P3, P4, P5, and P6, respectively. No image artifacts or contrast distortion in the spatial domain were observed in these particular measurements.

### Photothermal Decay Profile Evaluation

**Figure 7:**
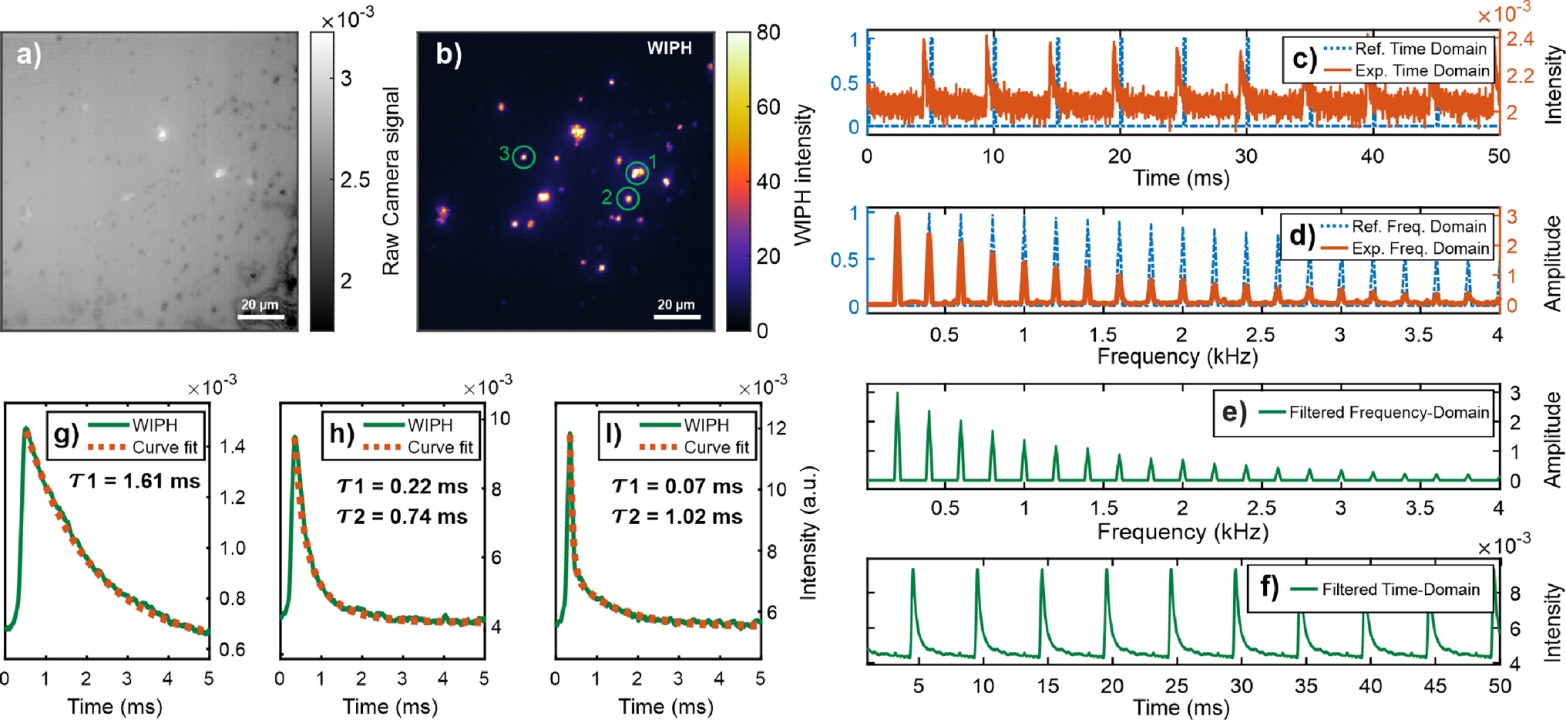
(a, b) Raw camera and WIPH images, respectively, of potassium ferricyanide microparticles. The evaluated particles are marked with a green circles. (c–f) Graphs showing the DFdLi filter steps during evaluation of particle 2 highlighted in (b). (g–i) Curve fits of thermal decay profiles, yielding thermal decay time constants of 1.61 ms for particle 1, 0.22 and 0.74 ms for particle 2, and 0.07 and 1.02 ms for particle 3.

The data presented in Figure 6a was also used to determine thermal decay profiles and time constants of single K-FeCy microparticles in 1-propanol (exp. E in Table 1). The results of this evaluation are shown in Figure 7, where Figure 7a,b represents the raw camera and WIPH images, respectively. Three particles of different sizes are marked with circles in the WIPH image. The time domain WIPH signals obtained by the camera pixels (Figure 7c) were filtered with the DFdLi (Figure 7c–f), employing a 200 Hz square wave with a 2% duty cycle as reference signal in order to minimize noise and recover the thermal decay profile (Figure 7f) by extracting all harmonics. The data processing shown in Figure 7c–f is from particle 2 (see Figure 7b). Figure 7g–i shows the thermal decay profiles recovered from particles 1–3, respectively.

The thermal decay time constant for particle 1 was obtained by fitting a power function *T*(*t*) ∝ *I*(*t*) = *I*_0_ + *A*_1_ exp ( – *t*/τ_1_) to the experimental data. For particles 2 and 3, the response was more complex, requiring two power functions *T*(*t*) ∝ *I*(*t*) = *I*_0_ + *A*_1_ exp ( – *t*/τ_1_) + *A*_2_ exp ( – *t*/τ_2_). Here, *T* is the instantaneous temperature, *t* is the time, *I* is the WIPH signal intensity, *I*_0_ is an offset parameter (corresponding to the initial temperature of the surrounding), *A*_1_ and *A*_2_ are the signal amplitudes, and τ_1_ and τ_2_ are the thermal decay time constants.^26^ The retrieved decay time constant for particle 1 was 1.61 ms, whereas particles 2 and 3 had values of 0.22 and 0.74, and 0.07 and 1.02 ms, respectively.

Since the decay time is defined as τ = *mC_s_*/*hS*, where *m* and *C_s_* are the mass and specific heat capacity of the absorber, and *h* and *S* represent the heat transfer coefficient and effective transfer surface area between the specimen and the surrounding, respectively,^26^ a decay measurement can, in principle, be used to determine the thermal properties of a microparticle-surrounding system.

### Widefield Mid-Infrared Photothermal Heterodyne Imaging of Lipids in a Living Cell

**Figure 8:**
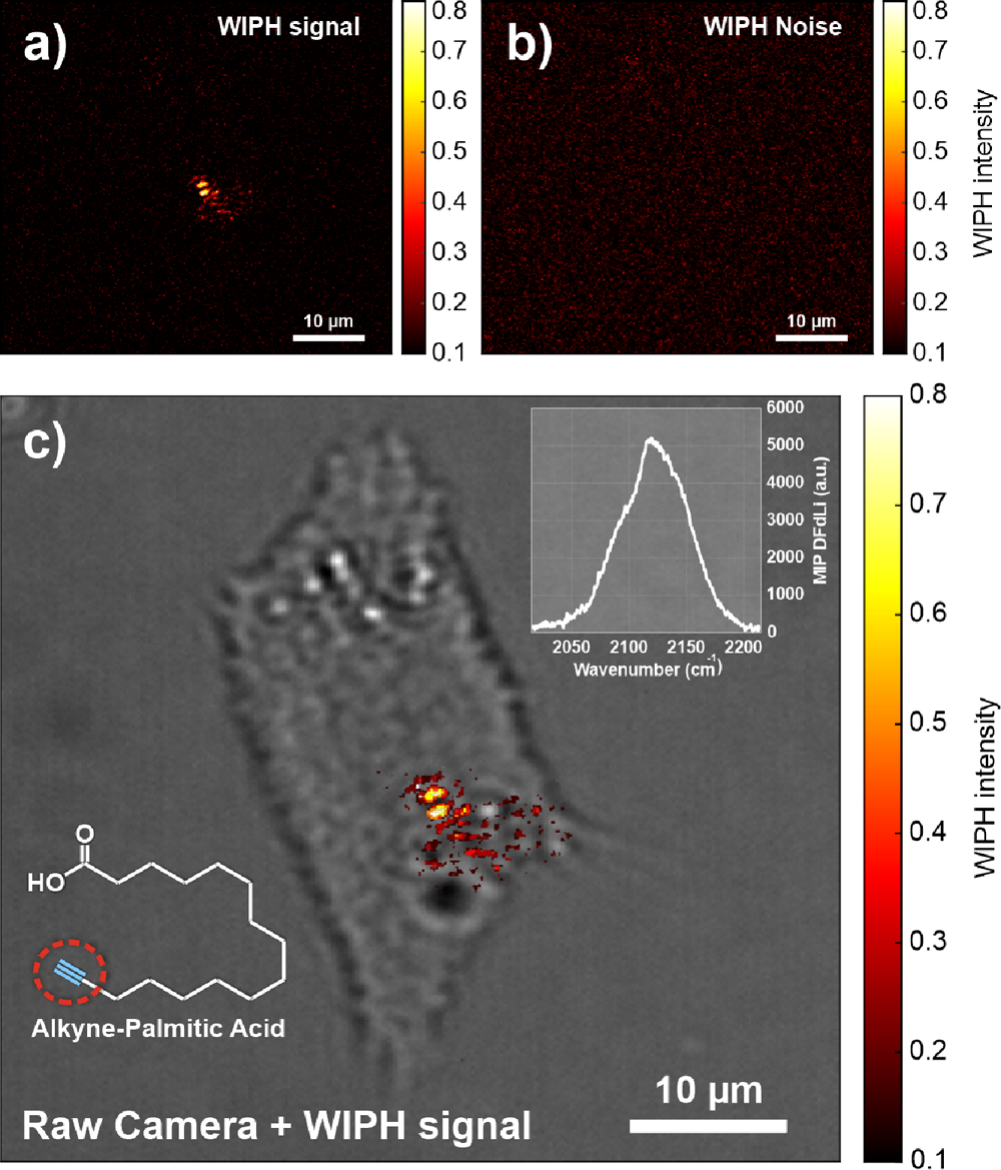
(a) WIPH image of a lipid droplet (alkyne-palmitic acid) in a living cell (IR modulation frequency 500 Hz). (b) WIPH image of the noise evaluated at 600 Hz. (c) Merged raw camera and WIPH images to spatially localize the lipid droplet in the 3T3-L1 cell. The insets show the chemical structure of alkyne-PA and the measured MIP spectrum of alkyne–palmitic acid in the cell-silent window (peak at 2120 cm^–1^).

In order to demonstrate the performance and potential of the WIPH technique, alkyne-tagged fatty acids were imaged in live cells (Figure 8), using the acquisition parameters shown in Table 1 (exp. F). To avoid thermal saturation of the target molecules in solution, the pump modulation signal was designed as a burst of pulses at 100 kHz modulated with a 500 Hz square wave, thus down-converting the modulation frequency to fit within the bandwidth of the camera.

Alkyne-PA is a free fatty acid tagged with an alkyne vibrational probe (see its chemical structure in the inset in Figure 8c) that exhibits absorption features in the cell-silent window. First, the MIP spectrum of alkyne-PA particles was recorded (inset in Figure 8c) using the photodetector and an IR pump modulation frequency of 2 kHz. As expected, a peak at 2120 cm^–1^, associated with the C≡CH stretching vibration, was found. Figure 8a displays the WIPH image demodulated at the IR modulation (500 Hz), clearly showing the lipid droplets containing alkyne-PA. The high chemical contrast with respect to the complex background stems from the fact that only the target molecules absorb in the cell-silent window. The WIPH noise level (Figure 8b) was evaluated at a frequency different from the IR pump modulation frequency and its harmonics (600 Hz in this case). Finally, Figure 8c presents the WIPH image merged with the brightfield image to show the location of the lipid droplet in the cell.

## Discussion

We have developed an ultrafast widefield photothermal heterodyne imaging system based on a novel digital frequency domain lock-in detection scheme, which allows (i) the use of cw probe light, thereby eliminating the need for synchronization between pump/probe modulation and camera shutter release, (ii) multiharmonic demodulation, and (iii) thermal decay measurements.

The WIPH microscope can be used to record the IR spectra of chemical compounds by scanning the pump laser wavelength (Figure 2), and for widefield imaging at a fixed IR laser wavelength (Figures 3–7). Once a data cube has been recorded, a suitable averaging time can be chosen (within the total acquisition time). In principle, hyperspectral imaging is also possible by acquiring the full IR spectrum with each camera pixel. However, since it takes 27 s to scan the EC-QCL wavelength range, this would yield a large amount of raw data (hundreds of Gb).

The system operates at a sensitivity level close to the brightfield shot-noise limit, for the ranges of probe power and integration time investigated, and in accordance with the theoretical description of heterodyne MIP (Figures 4 and 5). As expected, the SNR increases with the probe power, integration time, and number of demodulated harmonics. The inclusion of the higher harmonics in the demodulation improved the SNR by a factor of ∼2, since all MIP signal components were recovered (Figure 6). This was also demonstrated in a previous work using point-scan imaging.^15^

Based on the Abbe resolution equation *R*_*x*, *y*_ = λ/2NA, the theoretical diffraction limit of the current WIPH system is 411 nm. As confirmed with the help of the test target, the spatial coverage of one camera pixel, which is defined by the objective magnification (40×), the focal length of the tube lens (200 mm), and the pixel size (20 μm), is ∼500 nm. However, the effective WIPH spatial resolution is around 1 μm (Figure 4). This can be explained by the fact that, in practice, heat transport and thermal blurring deteriorate the MIP spatial resolution.^27^ Here, we used a relatively high IR pump power for maximum SNR in scattering field detection, which increases the thermal blurring effect. In addition, the spatial resolution was further compromised by the relatively large pixel size of the high-speed camera.

The WIPH instrument reaches unprecedented speed, as it enables imaging an area of 128 × 128 μm at 4000 frames/s while providing chemical contrast with an SNR of 5 (Figure 5). This is achieved by digital deconvolution of the time-resolved camera signal at each pixel and constitutes a significant advance in infrared imaging, pushing its limits toward faster and more sensitive detection in a wider field-of-view.^16−18^

In addition to the SNR improvement, another benefit of multiharmonic demodulation is that it allows to extract the sample thermal decay profiles for every camera pixel (Figure 7). The ability to record decay profiles is an important feature that has proven useful for instrument design and optimization, theory development, signal simulation, and target compound characterization.^15,26^

Here, it is demonstrated for K-FeCy microparticles that the thermal decay time decreases with particle size, most likely because the effective transfer surface area between the specimen and the surrounding increases. In general, K-FeCy exhibits longer thermal decay times than polymer beads of the same size,^26^ even when suspended in 1-propanol, which facilitates heat dissipation. K-FeCy crystals consist of inorganic molecules that are several times smaller than large organic polymer molecules containing many chemical bonds. Thus, crystalline K-FeCy has less degrees of freedom (molecular motions) to dissipate its thermal energy than amorphous polymer particles, such as PMMA, yielding a longer decay time.

The present WIPH imaging system is designed to operate within the IR cell-silent window (1900–2600 cm^–1^). The advantage of working in the cell silent region is that the main biological molecules contained in cells, and water, do not absorb in this spectral range. On the other hand, several small vibrational probes that can be attached to biomolecules do have distinct absorption features in the cell-silent window, which enables detection with high specificity and chemical contrast, undisturbed by the medium and the probe.^28^ Metal carbonyl, nitrile, cyanide, azides, alkynes, and deuterium are examples of vibrational probes already demonstrated for this purpose using Raman and IR absorption methods.^29−32^ Here, we successfully demonstrate, for the first time, detection of alkyne-tagged lipids using photothermal techniques by selective imaging of alkyne-PA in living 3T3-L1 cells (Figure 8). The quality of the final WIPH transmission image is still limited by a residual diffraction patter that depends on the focal plane position. This issue will be addressed in a future work.

The performance reached with the current WIPH system, including the capability of live cell analysis, suggests that it is suitable for in situ studies of dynamic biological processes at the cellular level in real time. Time-resolved imaging of cellular FFA uptake and protein-lipid interactions in the cell membrane^4^ is of particular interest, as it could elucidate how protein complexes control the internalization and storage of fatty acids in cells.^33,34^ Fatty acids and lipid droplets have pivotal roles in various functions of the body, including signaling to control cellular processes, such as inflammation, metabolism, as well as cardiovascular and neurodegenerative functions.^35,36^

The WIPH microscope presented here can be further improved. Since the DFdLi filter processes the raw data cube along the time dimension, the WIPH imaging system can be coupled to quantitative phase imaging techniques, which evaluate the data along the spatial dimensions. This could enhance the sensitivity for small structures, provide depth information, and deliver quantitative chemical information without calibration. A sensitivity improvement will also allow to use lower IR power, resulting in higher photothermal spatial resolution.

Importantly, WIPH-DFdLi imaging provides the possibility to simultaneously track more than one chemical compound in a sample by employing more than one pump laser, each using a different modulation frequency to be individually extracted by the DFdLi filter. Simultaneous, multispecies analysis with high speed cannot be performed by existing MIP techniques.

Finally, we believe that the DFdLi filter concept can be used to improve the performance of existing methods. In MC-PC^18^, for example, a transient phase-signal could be acquired instead of employing an on–off approach. The filter could even enable widefield detection in stimulated Raman scattering.

## Conclusions

This work describes an ultrafast widefield mid-infrared photothermal heterodyne imaging technique, where a novel digital frequency-domain lock-in filter was used to enable camera-based lock-in detection. The phase-insensitive extraction of multiple harmonics of the pump modulation frequency simplifies widefield MIP microspectroscopy, as it allows the use of cw probe light, thereby circumventing the need for elaborate synchronization schemes. The subsequent high SNR rivals the sensitivity typically achieved with heterodyne MIP and point scanning. The WIPH microscope is demonstrated by imaging K-FeCy particles in the infrared cell-silent window with up to 4000 frames/s at a signal-to-noise ratio of 5.52 and a spatial resolution of ≤1 μm in a 128 × 128 μm field of view. It is shown that multiharmonic demodulation leads to an SNR improvement and allows retrieving the thermal decay time constant characteristic of the analyzed sample. Finally, photothermal detection of alkyne-tagged fatty acids in living 3T3-L1 cells using the cell-silent spectral region is demonstrated for the first time. In a single platform, the developed WIPH technique simultaneously provides high chemical contrast, spatial resolution, and speed in a wide field of view. This constitutes a significant advancement of vibrational microspectroscopy and opens up for chemical imaging of fast processes in life science, medicine, and material science.
